# Encapsulation of Transforming Growth Factor-β3 in Poly(hydroxybutyrate-co-hydroxyvalerate) Nanoparticles for Enhanced Cartilage Tissue Engineering

**DOI:** 10.3390/ijms26114997

**Published:** 2025-05-22

**Authors:** Ana Isabel Rodríguez-Cendal, José Señarís-Rodríguez, María Piñeiro-Ramil, Loreto Cabarcos-Mouzo, María del Carmen Veiga-Barbazán, Rosa María Mejide-Faílde, Francisco Javier de Toro-Santos, Isaac Manuel Fuentes-Boquete, Silvia María Díaz-Prado

**Affiliations:** 1 Universidade da Coruña, Grupo de Investigación en Terapia Celular y Medicina Regenerativa, Departamento de Fisioterapia, Medicina y Ciencias Biomédicas, Facultad de Ciencias de la Salud, 15071 A Coruña, Spain; ana.rodriguezc@udc.es (A.I.R.-C.); jsenrod@yahoo.es (J.S.-R.); maria.pramil@udc.es (M.P.-R.); rosa.meijide.failde@udc.es (R.M.M.-F.); javier.toro@udc.es (F.J.d.T.-S.); i.fuentes@udc.es (I.M.F.-B.); 2 Fundación Pública Gallega de Investigación Biomédica, Grupo de Investigación en Terapia Celular y Medicina Regenerativa, Instituto de Investigación Biomédica A Coruña (INIBIC), Complexo Hospitalario Universitario de A Coruña (CHUAC), Servizo Galego de Saúde (SERGAS), 15071 A Coruña, Spain; 3 Universidade da Coruña, Grupo de Investigación en Terapia Celular y Medicina Regenerativa, Centro Interdisciplinar de Química e Bioloxía (CICA), 15071 A Coruña, Spain; 4 Servicio de Cirugía Ortopédica y Traumatología, Complexo Hospitalario Universitario de A Coruña (CHUAC), Servizo Galego de Saúde (SERGAS), 15071 A Coruña, Spain; 5 Universidade da Coruña, Grupo de Investigación en Bioingeniería Ambiental y Control de Calidad (BIOENGIN), Centro Interdisciplinar de Química y Biología (CICA), 15071 A Coruña, Spain; l.cabarcos@udc.es (L.C.-M.); m.carmen.veiga@udc.es (M.d.C.V.-B.); 6 Servicio de Reumatología, Complexo Hospitalario Universitario de A Coruña (CHUAC), Servizo Galego de Saúde (SERGAS), 15071 A Coruña, Spain; 7 Centro de Investigación Biomédica en Red de Bioingeniería, Biomateriales y Nanomedicina (CIBER-BBN), 28029 Madrid, Spain

**Keywords:** polyhydroxyalkanoate (PHA), regenerative therapy, drug delivery system (DDS), nanoparticles (NPs), poly(hydroxybutyrate-co-hydroxyvalerate) (PHBV)

## Abstract

Poly(hydroxybutyrate-co-hydroxyvalerate) (PHBV) is a naturally occurring biopolymer belonging to the polyhydroxyalkanoate (PHA) family. Due to its excellent properties (biocompatible, biodegradable, and non-toxic), this biopolymer is presented as a very suitable option for use in regenerative therapy as a drug delivery system (DDS). The protein encapsulated in this study is transforming growth factor β3 (TGF-β3), which plays a key role in the chondrogenic differentiation of mesenchymal stem cells (MSCs). The main objective of this work is to evaluate the efficacy of PHBV nanoparticles (NPs) produced from a dairy by-product (whey) as a DDS of TGF-β3 for cartilage regeneration and extracellular matrix (ECM) synthesis and to reduce the complications associated with multiple high doses of TGF-β3 in its free form. For this purpose, biopolymer cytotoxicity, factor release, cell viability, cell proliferation, and differentiation were analyzed. The results showed that the biomaterial purified with chloroform and ethanol, either by single or double precipitation, was not toxic to cells. A sustained release profile was observed, reaching its maximum around day 4. The TGF-β3 NPs promoted the differentiation of MSCs into chondrocytes and the formation of ECM. In conclusion, PHBV demonstrated its potential as an optimal material for DDSs in cartilage regenerative therapy, effectively addressing the key challenge of the need for a single delivery method to reduce complications associated with multiple high doses of TGF-β3.

## 1. Introduction

Osteoarthritis (OA) is a disorder that affects approximately 530 million people worldwide and is expected to increase in the coming years [1]. OA begins with the abnormal functioning of the metabolism of the joint tissue and is followed by physiological and anatomical changes such as inflammation, cartilage degradation, and the loss of normal joint function [2,3]. This joint disorder frequently affects women [4] as well as people with obesity [5]. OA is one of the ten most prevalent chronic diseases in developed countries. In Spain, it is known that almost a third of the population (29.35%) suffers from this condition in one or more areas of the body. The highest values are related to the lower back (15.52%) and the knee (13.83%) [6].

In 2022, a study endorsed by the main institutions focused on chronic pain in Spain, the Sociedad Española del Dolor (SED) and the Sociedad Española Multidisciplinar de Dolor (SEMDOR), showed that OA was one of the most commonly diagnosed causes of chronic pain in patients. It also pointed out that the treatments associated with this disease are often ineffective and, in some cases, do not alleviate the symptoms related to the pathology [7,8].

It is estimated that the annual direct and indirect costs associated with the treatment of OA are EUR 7.2 and EUR 4.6 billion [9]. In addition to being a social issue, it significantly impacts the country’s economy. Specifically in Spain, it was estimated in the past that the cost represented 0.5% of the country’s gross domestic product [10]. That means that approximately EUR 5 billion is spent annually on treatment, but 70% of affected patients are not satisfied with the management of their disease, and 50% continue to struggle with the related pain without finding any valid solution [11].

Education, weight control, exercise, and analgesics are some of the current treatments for OA, but they fail to stop the progressive destruction of cartilage associated with the disease [12]. OA was traditionally viewed as a passive degenerative joint disease related to long-term mechanical stress, but emerging viewpoints indicate that OA is an active and dynamic process [13]. Recently, this pathology has been reclassified as an inflammatory systemic disease with abnormal metabolic implications for chondrocytes. Articular cartilage consists of chondrocytes surrounded by an extracellular matrix (ECM) network of mainly proteoglycans and collagen. Chondrocytes maintain joint homeostasis in normal joints by modulating ECM synthesis and degradation. In osteoarthritic joints, pro-inflammatory cytokines such as interleukin 1-beta (IL-1β) and tumor necrosis factor (TNF-α) activate the nuclear factor-kappaB (NF-κB) signaling pathway that causes the systemic inflammation of all joint tissues, including the cartilage, synovial membrane, subchondral bone, and ligaments. Chondrocytes undergo various phenotypical changes that elevate cytokine expression (TNF-α, IL-1β, and interleukin 6 (IL-6)) and ECM degradative enzymes like matrix metalloproteinase (MMP) and aggrecanases. Such phenotypical changes result in cartilage destruction. The NF-κB molecule also activates nitric oxide (NO), cyclooxygenase 2 (COX-2), inducible nitric oxide synthase (iNOS), and prostaglandin E2 (PGE2), which promote catabolism and chondrocyte apoptosis [14,15,16].

A potential treatment for OA due to its protective effect is transforming growth factor β3 (TGF-β3), a protein necessary for different processes related to cell growth, development, and the differentiation of mesenchymal stem cells (MSCs) [17]. TGFβ3 is used for hyaline cartilage repair because it stimulates chondrogenesis and suppresses inflammatory factors such as interleukin 1 (IL-1) and TNF-α as well as MMPs [18]. The main drawback of this factor is that in its free form, there is rapid enzymatic degradation and an overdose in the tissue, which is why, in the present work, a drug delivery system (DDS) with nanoparticles (NPs) made of poly(hydroxybutyrate-co-hydroxyvalerate) (PHBV) is evaluated as a method of supplying it [19].

From a pharmacological point of view, the use of a DDS for the treatment of OA is a great advantage, as it improves the physical and chemical stability of the encapsulated active ingredients and increases bioavailability. This allows a much more controlled release of the drug, which can improve therapeutic efficacy by maintaining a constant concentration of the drug in the blood for a longer period of time and also decrease the side effects of treatment [20]. In addition, a DDS can be designed to penetrate certain drugs or proteins into specific tissues [21,22]. Nanocarriers commonly used as DDSs are exosomes, liposomes, micelles, and dendrimers, which are inorganic and polymeric NPs [23].

Among the biomaterials used in the literature, we find polyhydroxyalkanoates (PHAs). They are a family of polyesters, biocompatible and biodegradable, biosynthesized by a wide variety of microorganisms, mainly bacteria [24,25]. They were discovered in 1926 by Lemoigne, M., when he observed the bioaccumulation of these materials in the bacteria of the genus *Bacillus megaterium* when cultured under controlled fermentation conditions [26]. They are a group of polymers that can be obtained from more than 150 monomers [27], which allows the polymer to be custom-designed, resulting in changes in polymer properties that can range from hard and fragile to soft and elastomeric. The traditional synthesis of PHAs uses pure cultures and synthetic substrates. However, a new and much more economical method using mixed cultures of microorganisms and industrial waste or by-products as substrates, such as whey, is being studied. This process recovers the waste product and also helps solve an environmental problem [28].

The most widely used PHA biopolymer is polyhydroxybutyrate (PHB) because it is very similar to polypropylene (PP), but its fragility is what makes it difficult to replace PP. The incorporation of hydroxyvalerate (HV) monomers into the PHB biopolymer causes the material to lose its breakability, making it more flexible and resistant [29]. For that reason, PHBV copolymers are more useful in the development of DDSs than PHB homopolymers [30]. PHBV has been used in the controlled release of anticancer drugs, in photodynamic therapy for cancer, in the treatment of Parkinson’s disease, and in trans-dermal therapies for skin conditions such as psoriasis and aging, among other applications [31]. It should be noted that purification of the biopolymer is necessary to obtain endotoxin levels below those established by the Food and Drug Administration (FDA) [32].

## 2. Results

### 2.1. Biopolymer Cytotoxicity

**Figure 1:**
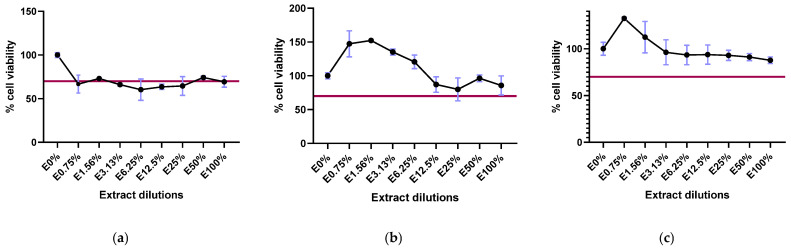
Cell viability (%) in the presence of different concentrations of extract. (**a**) Biomaterial purified with peroxide and ethanol at 70%; (**b**) biomaterial purified by a single precipitation with chloroform and ethanol at 86%; (**c**) biomaterial purified by a second precipitation with chloroform and ethanol at 86%. The red line in the graphs marks the 70% viability required by the ISO 10993-5 standard.

In the cytotoxicity study, concentrations capable of reducing cell viability by at least 30% were considered harmful. For the first biomaterial, which was purified with peroxide and 70% ethanol, cell viability with the different extracts ranged from 60.30 ± 12.30% to 74.24 ± 1.76% (Figure 1a). Although it was borderline, it was considered a cytotoxic material. However, for the biomaterial purified after a single precipitation with chloroform and 86% ethanol, the cell viability for the 100% undiluted extract was 85.69 ± 14.17% (Figure 1b). Finally, the biomaterial purified after a second precipitation using chloroform and 86% ethanol was 87.71 ± 3.58% (Figure 1c). The last two biopolymers were not considered cytotoxic. The endotoxin levels obtained with the last two methods of purification mentioned were satisfactory, with values below 1 EU/g of PHBV, complying with the standards established by the FDA.

### 2.2. Factor Release Study

**Figure 2:**
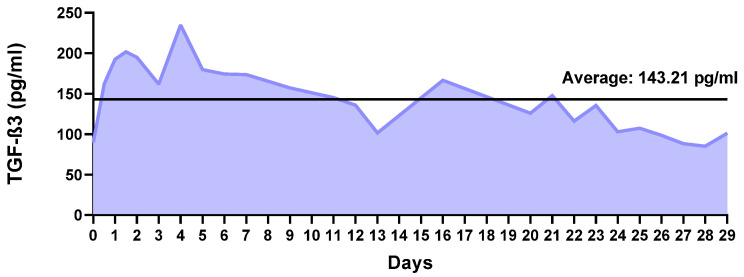
Non-cumulative transforming growth factor β3 (TGF-β3) release profile over 29 days from the nanoparticles.

Figure 2 shows the non-cumulative release of TGF-β3 from the NPs over 29 days. A progressive increase in TGF-β3 concentration was observed until day 4, when it reached a maximum value close to 235 pg/mL. From day 5 onward, the concentration of protein released into the medium gradually decreased until day 16, when it again reached a higher value of 167 pg/mL. A fluctuating release was observed from then on but with a general trend toward a lower release. On average, the daily release was approximately 143.21 pg/mL, with a standard deviation of 41.07 pg/mL.

### 2.3. Biocompatibility of the Nanoparticles

**Figure 3:**
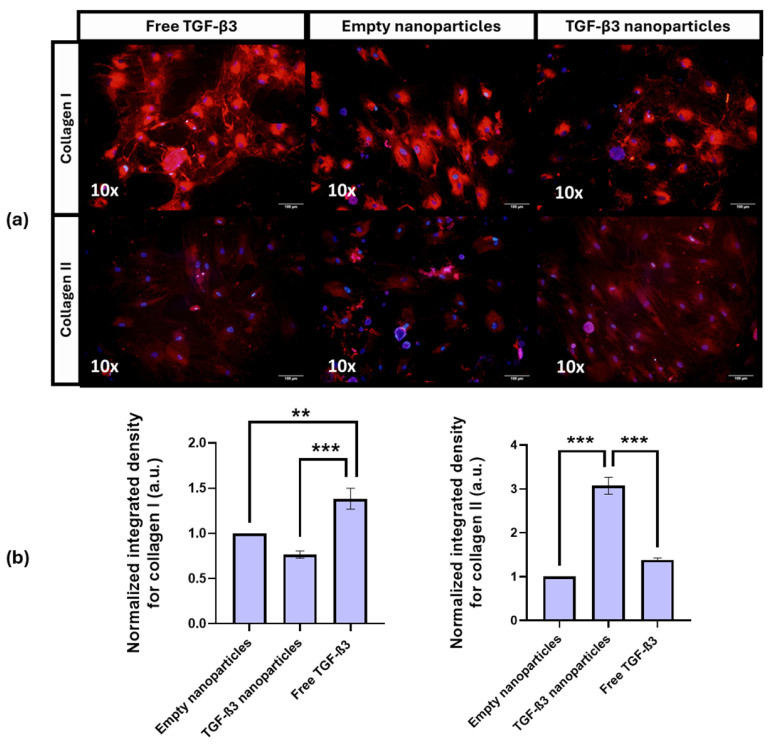
(**a**) Representative immunofluorescence images of cells cultured for 21 days under different conditions: treatment with free transforming growth factor β3 (TGF-β3) added twice a week, empty nanoparticles, and TGF-β3 nanoparticles added at the beginning of the experiment. Immunofluorescence analysis was performed using anti-collagen type II ab34712 (Abcam, Cambridge, UK) and anti-collagen type I ab138492 (Abcam, Cambridge, UK) as primary antibodies and rabbit anti-IgG (H + L) and Alexa Fluor™ 594 cross-adsorbing antibody (Thermo Fisher, Waltham, MA, USA) as secondary antibodies. Counterstaining was performed with Bisbenzimide Hoechst 33342 (Merck, Darmstadt, Germany). Scale bar represents 100 μm; (**b**) quantification of collagen I and II expression based on the normalized integrated density in arbitrary units (a.u.) under the same conditions. Statistical significance was assessed using one-way ANOVA, followed by Tukey’s test. Significance levels were defined as follows: *** *p* < 0.01; ** *p* < 0.05.

In the two dimensions (2D) differentiation experiment (Figure 3a), all immunofluorescence images shown in the Supplementary Figure S1 were analyzed using ImageJ. The normalized integrated density was calculated, and values were set relative to the negative control with empty NPs (normalized to 1; Figure 3b).

The results showed that collagen I (COL I) expression was significantly higher in cells treated with free TGF-β3 in the positive control (1.38 a.u. ± 0.12 a.u.) than in those treated with TGF-β3 NPs (0.77 a.u. ± 0.04 a.u.; *p* < 0.0026) and empty NPs (*p* = 0.0253). No significant difference was found between the empty NP group and the TGF-β3 NP group (*p* = 0.1447). For collagen II (COL II), the TGF-β3 NPs induced the highest expression (3.07 ± 0.19 a.u.), which was significantly higher than those of both the positive control group with free TGF-β3 (1.38 ± 0.04 a.u., *p* < 0.0001) and the empty NP group (*p* = 0.0001). No significant difference was observed between the empty NP group and the positive control with free TGF-β3 (*p* = 0.1437).

These results demonstrate that TGF-β3 delivered via NPs significantly enhances COL II expression and reduces COL I levels, suggesting a favorable shift toward chondrogenic differentiation and reduced fibrotic response.

**Figure 4:**
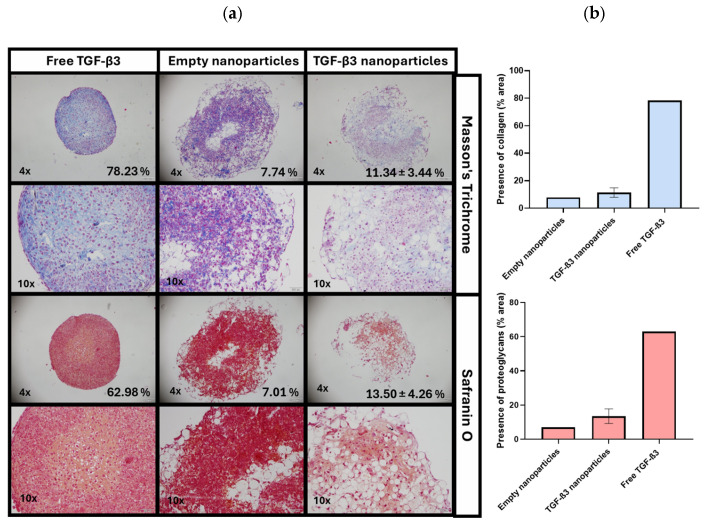
(**a**) Representative histological staining of Masson’s Trichrome and Safranin O of micromasses cultured for 21 days under different conditions: treatment with free transforming growth factor β3 (TGF-β3) added twice a week, empty nanoparticles, and TGF-β3 nanoparticles added at the beginning of the experiment. Scale bars represent 200 μm in the 4× images and 100 μm in the 10× images. (**b**) Calculated percentage of area related to collagen and proteoglycan formation under the same conditions. Statistical analysis was not conducted due to the limited number of replicates in the control groups.

In the three dimensions (3D) differentiation experiment, micromass sections were stained with Masson’s Trichrome (MT) to evaluate cell morphology and collagen deposition and with safranin O (SO) to assess the proteoglycan content. Representative images of both stainings are shown in Figure 4a, where MT staining (upper row) highlights nuclei in purple, cytoplasm in pink, and collagen in blue, while SO staining (lower row) indicates proteoglycans through orange–red metachromasia. Complete sets of images for all the experimental conditions are provided in Supplementary Figure S2. Quantitative analysis of the stained areas for collagen and proteoglycans is presented in Figure 4b.

A good percentage of the colored area related to collagen formation was observed in the positive control with free TGF-β3 (78.23%). In contrast, micromasses treated with TGF-β3 NPs showed significantly reduced collagen staining (11.34 ± 3.44%) due to the presence of NPs, which interfered with matrix formation by reducing cell interaction. In the empty NPs, almost no blue staining was seen (7.74%).

The presence of proteoglycans was evident in the micromass treated with free TGF-β3 (62.98%), while the TGF-β3 NP group exhibited a lower percentage (13.50 ± 4.26%). The absence of proteoglycan synthesis was evident in those treated with empty NPs (7.01%). Given the limited number of replicates in the control groups, statistical analysis was not feasible.

**Figure 5:**
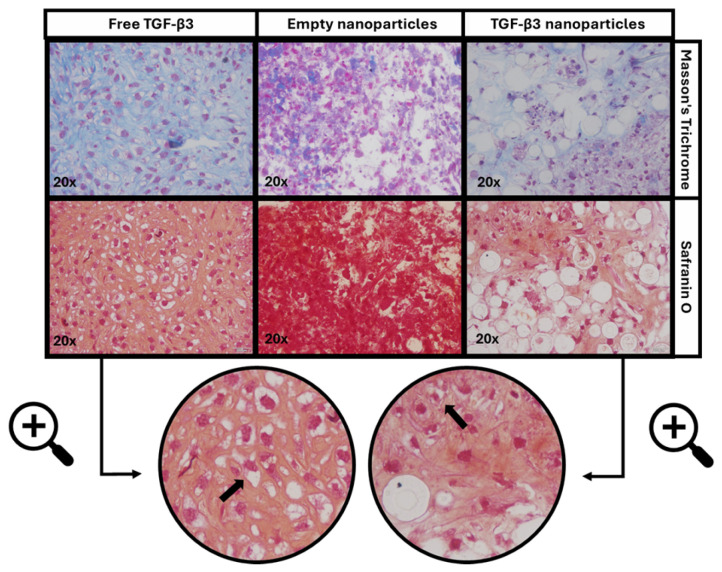
The pericellular lacuna of cells in micromasses (marked with arrows in the figure) cultured for 21 days under different conditions: treatment with free transforming growth factor β3 (TGF-β3) added twice a week, empty nanoparticles, and TGF-β3 nanoparticles added at the beginning of the experiment. Scale bar represents 50 μm.

Pericellular lacuna can also be observed in those micromasses treated with free TGF-β3 and TGF-β3 NPs (Figure 5).

### 2.4. Generation of Chondrogenic Constructs

**Figure 6:**
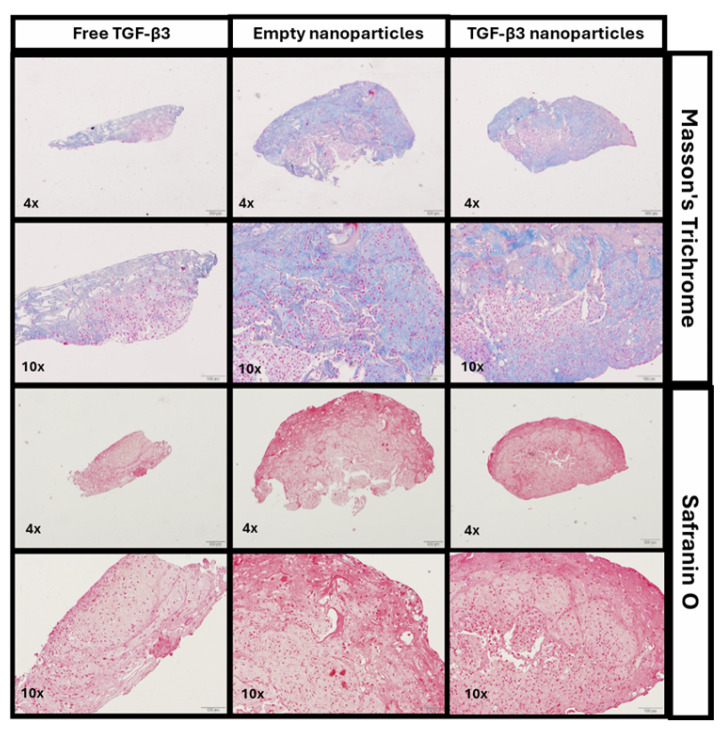
Representative histological Masson’s Trichrome and Safranin O staining of chondrogenic constructs cultured for 30 days under different conditions: treatment with free transforming growth factor β3 (TGF-β3) added twice a week, empty nanoparticles, and TGF-β3 nanoparticles added at the beginning of the experiment. Scale bars represent 200 μm in the 4× images and 100 μm in the 10× images.

The results for the constructs showed that the cells were distributed and expanded efficiently along the scaffold, favoring homogeneous regeneration. Furthermore, it was observed that the number of cells selected was adequate to completely cover the collagen I (Col I) scaffold, ensuring an even distribution over the entire surface. Representative histological images stained with MT and SO are shown in Figure 6, corresponding to the full set of images available in Supplementary Figures S3 and S4.

The MT staining revealed that the constructs in contact with the TGF-β3 NPs showed areas with more intense blue staining, although the differences were minimal compared with the positive control construct (free TGF-β3) and the negative one (empty NPs). This was mainly because the construct was made of Col I.

On the other hand, in the SO staining, there was a notable difference between the positive control with free TGF-β3 and the TGF-β3 NP groups compared with the negative control with empty NPs, suggesting a greater presence of proteoglycans in the first ones.

### 2.5. Ex Vivo Repair Model

**Figure 7:**
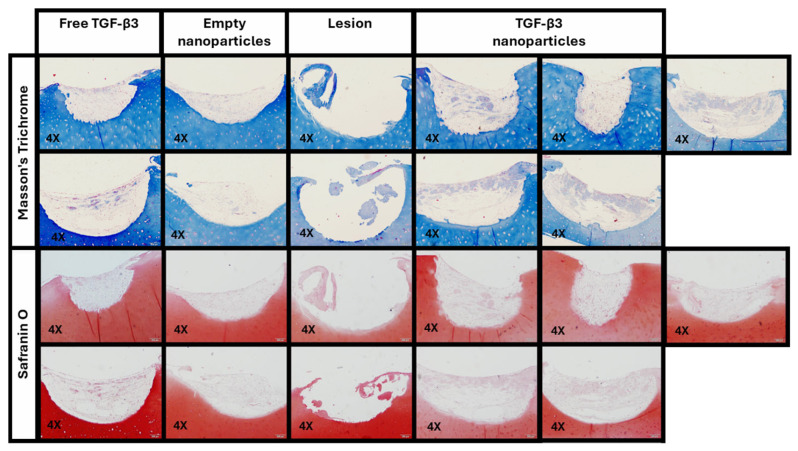
Histological staining with Masson’s Trichrome and Safranin O performed on the ex vivo repair model cultured for one and a half months under different conditions: treatment with free transforming growth factor β3 (TGF-β3) added twice a week, empty nanoparticles, and TGF-β3 nanoparticles added at the beginning of the experiment. Scale bars represent 200 μm.

In the ex vivo repair model, the quality of the repair tissue was evaluated using a modified version of the ICRS II semi-quantitative scoring system, considering only six parameters that can be reliably assessed in this type of model through MT and SO staining (Figure 7).

**Figure 8:**
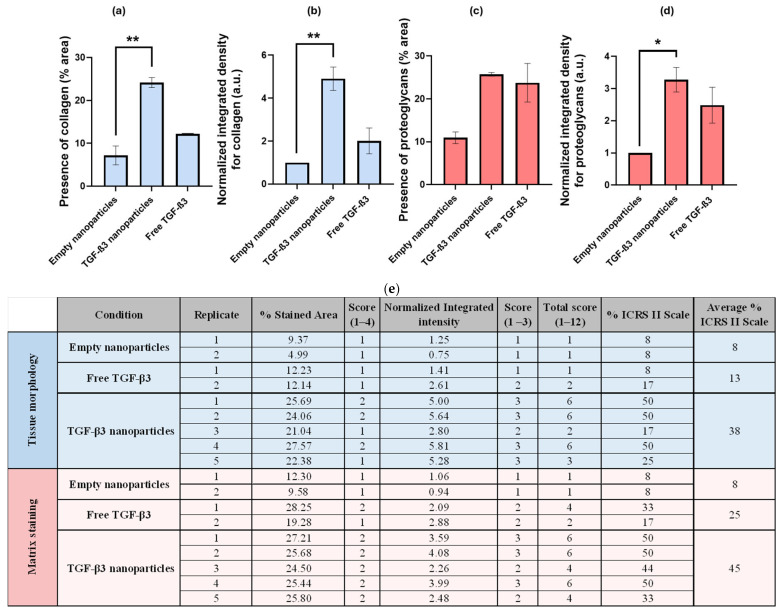
(**a**) Calculated percentage of area (%) related to collagen formation for the following conditions: treatment with free transforming growth factor β3 (TGF-β3) added twice a week, empty nanoparticles, and TGF-β3 nanoparticles added at the beginning of the experiment; (**b**) quantification of the normalized integrated density related to collagen formation in arbitrary units (a.u.) under the same conditions; (**c**) calculated percentage of area (%) related to proteoglycan formation under the same conditions; (**d**) quantification of the normalized integrated density related to proteoglycan formation in arbitrary units (a.u.) under the same conditions; (**e**) calculation of the final combined score, ranging from 1 to 12, that was used to estimate the degree of tissue repair according to the ICRS II criteria. Statistical analysis was performed using the Mann–Whitney U test, followed by Dunn’s multiple comparisons test. Significance levels were defined as follows: ** *p* < 0.05; * *p* < 0.1.

The first parameter to be assessed was tissue morphology, where the presence and distribution of collagen in the lesion were analyzed. Lower values were associated with a disorganized display of collagen fibers, while higher values reflected greater compactness and the homogeneous distribution of this protein. A significantly higher percentage of collagen area was observed in the group treated with TGF-β3 NPs (24.15 ± 1.16%) than in the empty NP group (7.18 ± 2.19%; *p* < 0.0491) and the group treated with free TGF-β3 (12.18 ± 0.04%; *p* < 0.3799) (Figure 8a). In addition, the normalized integrated density for collagen was significantly higher in the TGF-β3 NP group (4.91 ± 0.54 a.u.) than in the empty NP (*p* < 0.0491) and the free TGF-β3 groups (2.01 ± 0.60 a.u.; *p* < 0.3799), indicating a greater intensity of collagen staining per unit area (Figure 8b).

The second parameter measured was matrix staining, which assessed the proteoglycan content based on the presence or absence of metachromasia. In the SO staining, it was observed that the group treated with TGF-B3 NPs showed a similar percentage area of proteoglycans (25.72 ± 0.44%) to that treated with free TGF-β3 (23.76 ± 4.49%, *p* > 0.9999) but more than that treated with empty NPs (10.94 ± 1.36%, *p* < 0.1486) (Figure 8c). The normalized integrated density for proteoglycans was also significantly higher in the TGF-β3 NP group (3.28 ± 0.38 a.u.) than in the empty NP group (*p* < 0.0781) but was similar to that in the free TGF-β3 group (2.48 ± 0.56 a.u.; *p* > 0.9999) (Figure 8d).

All these results were processed as described in the methodology and converted into a unified score related to the ICRS II scale. Therefore, the TGF-β3 NP group achieved an average ICRS II rating of 38% for tissue morphology and 45% for matrix staining, while the free TGF-β3 group scored 13% and 25%, and the empty NP group scored 8% for both parameters (Figure 8e).

**Figure 9:**
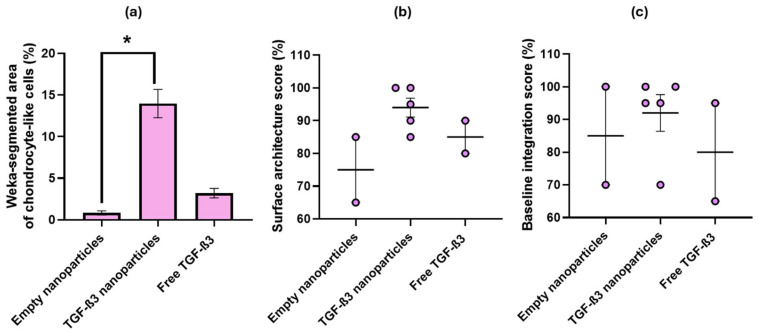
(**a**) Percentage of the area (%) segmented as chondrocyte-like cells using the Trainable Weka Segmentation tool under different conditions: treatment with free transforming growth factor β3 (TGF-β3) added twice a week, empty nanoparticles, and TGF-β3 nanoparticles added at the beginning of the experiment; (**b**) scores as a percentage for the surface architecture (%) under the same conditions; (**c**) scores as a percentage for baseline integration (%) under the same conditions. Significance level was defined as follows: * *p* < 0.1.

The third parameter was cell morphology. Oval cells, which showed a pericellular lacuna typical of hyaline cartilage, had higher values, while more elongated cells, characteristic of fibrocartilage, had lower values. In the group treated with TGF-β3 NPs, a significantly higher proportion of cells with typical hyaline cartilage morphology was observed by the Trainable Weka Segmentation tool (13.98 ± 1.70%) than in the empty NP (0.82 ± 0.25%, *p* < 0.0553) and the free TGF-β3 (3.20 ± 0.57%, *p* < 0.4719) groups (Figure 9a). Regarding chondrocyte clustering, no isogenic groups were detected in the neo-tissue, so the value assigned in all cases was 100%. The surface architecture was positively assessed—the smoother the surface, the fewer the disruptions. In the case of the TGF-β3 NP group, the average was 95.00 ± 2.74%; for the free TGF-β3 group, it was 85.00 ± 5.00%; and for the empty NP group, it was 75.00 ± 10.00% (Figure 9b). Finally, baseline integration was assessed considering the lesion but not the underlying bone. In the case of the TGF-β3 NP group, the average was 93.00 ± 5.83%; in the free TGF-β3 group, it was 80.00 ± 15.00%; and in the empty NP group, it was 85 ± 15.00% (Figure 9c).

The final scores, related to the ICRS II scale and therefore to the percentage of repair calculated based on all previously evaluated parameters, are shown in Table 1.

**Figure 10:**
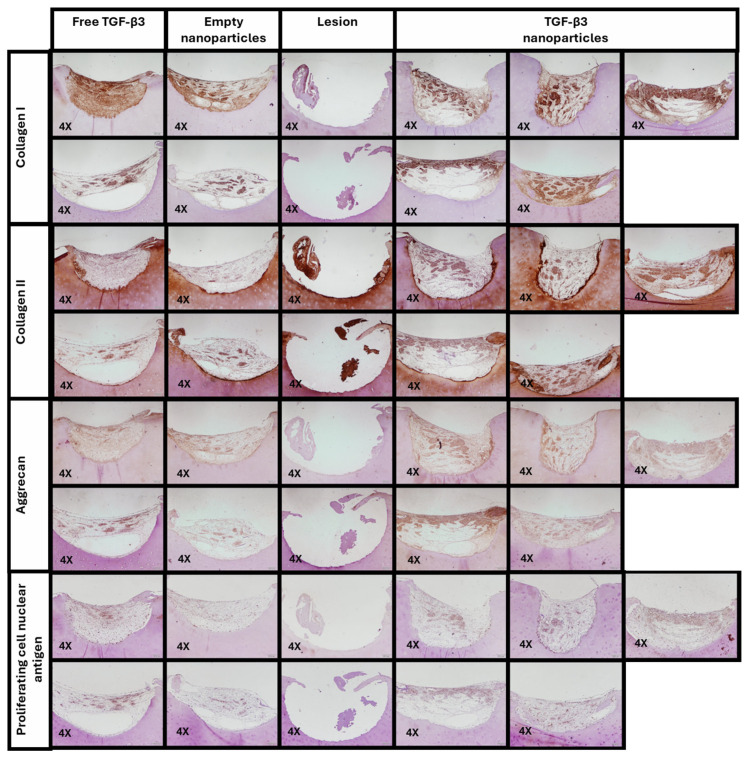
Immunohistochemical staining for collagen I, collagen II, aggrecan, and proliferating cell nuclear antigen for the ex vivo repair model cultured for one and a half months under different conditions: treatment with free transforming growth factor β3 (TGF-β3) added twice a week, empty nanoparticles, and TGF-β3 nanoparticles added at the beginning of the experiment. Scale bars represent 200 μm.

Immunohistochemical staining was also performed for COL I, COL II, aggrecan (ACAN), and the proliferating cell nuclear antigen (PCNA) to study the viability of the cells and the formation of ECM on the biomaterials (Figure 10).

**Figure 11:**
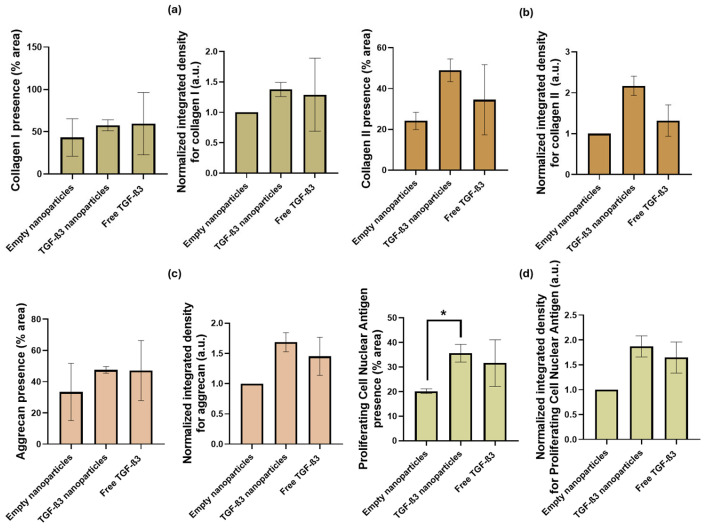
(**a**) Percentage area and normalized integrated density related to collagen I presence under the following conditions: treatment with free transforming growth factor β3 (TGF-β3) added twice a week, empty nanoparticles, and TGF-β3 nanoparticles added at the beginning of the experiment; (**b**) percentage area and quantification of the normalized integrated density related to collagen II presence under the same conditions; (**c**) percentage area and quantification of the normalized integrated density related to aggrecan presence under the same conditions; (**d**) percentage area and quantification related to proliferating cell nuclear antigen presence of the normalized integrated density under the same conditions. Significance level was defined as follows: * *p* < 0.1.

Immunohistochemical staining for COL I (Figure 11a) showed a generally strong signal, primarily attributed to the scaffold structure itself, as this collagen type is the primary component of the scaffold used to support tissue regeneration in this project. The positive control with free TGF-β3 (59.59 ± 36.78%) presents a slightly increased COL I-expressed area compared to the group treated with TGF-β3 NPs (57.60 ± 6.52%). The group with the least COL I expression was the empty NP group (43.29 ± 22.14%). The normalized integrated density was also similar in the free TGF-β3 group (1.29 ± 0.60 a.u.) and the TGF-β3 NP group (1.38 ± 0.12 a.u.) but was higher than in the empty NP group. No statistical differences were significant (*p* > 0.9999 in all cases).

For COL II expression (Figure 11b), an increased staining area was observed in the TGF-β3 NP group (48.95 ± 5.53%) compared with the positive control with free TGF-β3 (34.50 ± 17.17%; *p* < 0.8847) and the negative control with the empty NPs (24.13 ± 4.22%; *p* < 0.2662). The amount of this protein in the positive control with free TGF-β3 and the negative one with empty NPs was quite similar (*p* > 0.9999). The normalized integrated density was slightly higher in the TGF-β3 NPs group (2.17 ± 0.23 a.u.) than in the free TGF-β3 group (1.32 ± 0.38 a.u.; *p* < 0.4494) and the empty NP group (*p* < 0.1085). The normalized integrated density of this protein in the positive control with free TGF-β3 and the negative one with empty NPs was also similar (*p* > 0.9999).

In terms of ACAN (Figure 11c), the positive control with free TGF-β3 (47.16 ± 19.23%) presented the same area of expression as the group treated with TGF-β3 NPs (47.57 ± 2.17%) and was more than in the empty NP group (33.40 ± 18.30%). No statistical differences were significant (*p* > 0.9999 in all cases). The normalized integrated density was also similar in the free TGF-β3 group (1.45 ± 0.31 a.u.) and the TGF-β3 NP group (1.68 ± 0.16 a.u.; *p* > 0.9999) but higher than in the empty NP group (*p* < 0.3191).

Finally, PCNA staining (Figure 11d) revealed a significantly greater cell proliferation in the positive control with TGF-β3 NPs (35.61 ± 3.61%) and free TGF-β3 (31.60 ± 9.47%; *p* > 0.9999) than in the empty NP (20.17 ± 3.97%; *p* < 0.0974) group. The positive control with free TGF-β3 also had a higher expressed area than the empty NP group (*p* < 0.6037). The normalized integrated density was also similar in the TGF-β3 NP group (1.87 ± 0.21 a.u.) and the free TGF-β3 group (1.65 ± 0.31 a.u.; *p* > 0.9999) but higher than in the empty NP group (*p* < 0.1207). The positive control with free TGF-β3 also had higher normalized integrated density than the empty NP group (*p* < 0.4324).

## 3. Discussion

In tissue engineering and biomedicine, the materials must have characteristics that make them harmless to the human body. PHAs are well known for being biodegradable, biocompatible, and non-toxic. Cytotoxicity is a key parameter to assess when developing a new material. In the literature, several studies using PHBV with different percentages HV monomer were tested. Variations in HV content can affect both the material’s structure and its toxicity. For a 2% HV material, the cytotoxic potential of the NPs was investigated in a colon cancer cell line (HT-29), and the results of two different investigations showed that there was no negative effect related to this biomaterial [33,34]. In another trial, they tested a biopolymer with the same composition, and in this case, on a human epithelial cell line (A549), the results also showed relatively high biocompatibility [35]. In this project, the PHBV tested had a percentage of 12% HV. The cell line used in this case was a human keratinocyte cell line (HaCaT). The results showed that two of the methods of purification used with the materials were highly biocompatible, as the cell viability obtained for a 100% extract was very high. PHBV with the same monomer concentrations for cervical (HeLa) and ovarian (SKOV-3) cancer lines [36], as with A549 lines, proved to also be biocompatible in other studies [37].

Synthesized materials can be used in tissue engineering in different forms, depending on the study’s objectives. In this research, PHBV was selected to develop a DDS in the form of NPs. This system not only protects the drug or protein but also, in cases of sustained release, minimizes the risk of potential toxic effects associated with overdose, ensuring safer and more controlled delivery. The main procedures for efficiently loading the bioactive compound into the nanocarrier system are covalent bonds, electrostatic interactions, and encapsulation, where the internal hollow cavities of the structure are used as storage space for the drug. The loading of hydrophilic compounds, such as TGF-β3, into predominantly hydrophobic polymers is usually achieved by double emulsion–evaporation, complex coacervation, or nanoprecipitation [38]. In this work, the method used was the first one mentioned above.

TGF-β3 was chosen as the encapsulated molecule because it has been shown that growth factors belonging to the TGF superfamily can promote chondrogenesis in MSCs in vitro and in vivo. These molecules can act indirectly through membrane receptors and signaling pathways or directly by controlling transcription. Among the three existing isoforms of TGF-β (1,2,3), TGF-β3 was chosen because it is involved in the entire life cycle of chondrocytes, including their proliferation, migration, differentiation, and death.

Although TGF-β3 has both inducing and inhibitory effects, it is considered more pro-chondrogenic than TGF-β1. However, high doses of TGF-β3 can cause adverse effects, such as fibrosis, synovitis, pannus formation, cartilage erosion, and osteophyte formation in both cartilaginous and non-cartilaginous tissues [17]. That is one reason why, in this study, a DDS was proposed to address this issue by providing a more sustained release of the protein.

The next step was to analyze the release profile of the protein. In a study using PHBV with 3% HV and insulin as a hydrophilic drug, an initial burst release was observed, followed by a gradual release pattern that reached 63.2% over 27 days. Of this amount, 19% was released at a relatively rapid rate over 24 h, while the remainder was released gradually in a stationary phase [39]. In another case, where the NPs produced had 12% HV, most of the quercetin drug release occurred in the first 5 h of water immersion. At 15 days (360 h), the signal obtained was equivalent to that of the empty NPs, suggesting complete release. It should be noted that the amount of drug released was low, as it is related to the low water solubility of the compound [40]. In our experiment, the same concentration of HV was used, and the results showed higher releases on day 4 but with sustained release over time.

Controlled protein release aims to enhance cell viability, proliferation, and differentiation in various culture systems, including monolayer cultures, micromasses, and biomaterial scaffolds. Micromasses and ex vivo models are particularly useful for investigating the molecular mechanisms that regulate chondrogenesis [41].

In our study, monolayer cultures supported the differentiation of MSCs into chondrocytes, as evidenced by the increased expression of COL II and the reduction in COL I levels. In micromass cultures, despite some interference from the NPs, both collagen and proteoglycan deposition were still observed. In the ex vivo repair model, COL II expression was higher in the TGF-β3 NP group, and COL I, ACAN, and PCNA levels were similar to those in the group treated with free TGF-β3. Although in several parameters the values were comparable rather than superior, it is important to note that the encapsulated TGF-β3 was administered only once at the beginning of the experiment, whereas the free TGF-β3 was applied repeatedly to maintain the same concentration. This suggests that the DDS provided by the NPs system may be sufficient to achieve similar outcomes with a simplified dosing regimen. Other studies have explored the use of NPs to address similar challenges, employing biopolymers such as alginate microspheres coated with hyaluronic acid hydrogels [42], alginate alone, and chitosan [19].

Furthermore, using the ICRS II scale, we confirmed that all cartilage repair parameters showed better outcomes in cases where TGF-β3 NPs were present.

## 4. Materials and Methods

### 4.1. Synthesis of Poly(hydroxybutyrate-co-hydroxyvalerate) and Nanoparticle Formation

The production of PHBV and the formation of NPs by double emulsion–evaporation were carried out in collaboration with the Environmental Bioengineering and Quality Control Research Group of the University of A Coruña. In this case, the biopolymer was obtained from an industrial by-product, whey. The composition of the biomaterial was approximately 88% hydroxybutyrate (HB) and 12% HV. Three different purification methods were tested: one using peroxide and 70% ethanol, one with chloroform and 86% ethanol with one precipitation, and a third method with two precipitations using the same solvents. The method selected for producing the biomaterial in this project was that with chloroform and 86% ethanol with one precipitation.

### 4.2. Isolation and Extraction of Mesenchymal Stem Cells

The MSCs used in this project were isolated from bone marrow (BM) samples obtained from donors who had generally undergone hip and knee replacement surgery. All samples were provided by the Tissue Bank (A Coruña, Spain). It should be noted that all donors who participated in the study signed the informed consent form.

To isolate the cells from the BM samples, approximately 10 mL of Dulbecco’s Modified Eagle’s Medium (DMEM; Gibco, Paisley, UK), supplemented with 10% fetal bovine serum (FBS; Paisley, UK) and 1% penicillin/streptomycin (P/S; Gibco, Paisley, UK), was added first to the sample bottle. This liquid was passed through a 60 µm needle filter and centrifuged at 433 g for 5 min (Beckman Coulter Allegra X-22R, Beckman Coulter, Palo Alto, CA, USA). The supernatant was removed by decanting, and the cells were resuspended in approximately 5 mL of DMEM containing 20% FBS and 1% P/S. The content was added to a 25 cm^2^ culture plate (Costar Corning, Corning, NY, USA). The cells were placed in a 37 °C incubator with a humidified atmosphere containing 5% carbon dioxide (CO_2_) (CO_2_ incubator Sanyo MCO-18AIC UV, Sanyo Electric, Osaka, Japan). The first medium change was not performed for at least 48 h.

For the hip samples, cell extraction was carried out by washing the cancellous bone of the femoral head by injecting the DMEM culture medium with 5% FBS and 1% P/S using a 20 mL syringe and a wide bevel needle. After extraction, the culture medium with the cells was treated in the same way as the BM samples.

The culture plates with the seeded cells were changed with fresh DMEM culture medium containing 20% FBS and 1% P/S twice a week. Subcultures were performed when cell confluence reached 80–90%. To ensure the presence of MSCs only, a pre-plating technique was used.

### 4.3. Biopolymer Cytotoxicity

A cytotoxicity evaluation was performed following ISO 10993-5:2009 [43] guidelines for the in vitro biological assessment of medical devices. A human keratinocyte cell line (HaCaT) was thawed and expanded for the assays. Approximately 24 h before the experiment, 10,000 cells per well were seeded into 96-well plates (Costar Corning, Corning, NY, USA).

At the same time, following the recommendations of ISO 10993-12:2021 [44], an area of 6 cm^2^ was cut out of the PHBV material and then placed in contact with 1 mL of DMEM with 10% FBS and no phenol red. This process was also performed for two negative controls, one consisting of a Col I biomaterial and another with only the culture medium. The extraction was performed in an Eppendorf tube (Eppendorf, Hamburg, Germany) for 24 h at 37 °C with shaking.

After 24 h from the start of the extraction, 100 μL of the extracts in different percentages (from 100% to 0.75%) were added to the wells with the semi-confluent cells. As a negative control, a 100% extract from the Col I biomaterial was used, and as a positive control, sodium lauryl sulfate (Scharlab, Barcelona, Spain) was used at concentrations ranging from 5 mg/mL to 0.078 mg/mL. After 24 h of culture with the extracts, 10 μL of 3-(4,5-dimethylthiazol-2-yl)-2,5-diphenyltetrazolium bromide (MTT) reagent (Roche, Mannheim, Germany) was added and incubated for 4 h at 37 °C. Then, 100 μL of the solubilizing solution was added and incubated overnight at 37 °C.

The next day, absorbance was measured at 570 nm in a spectrophotometer (Infinite^®^ 200 PRO NanoQuant, Tecan, Männedorf, Switzerland).

### 4.4. Factor Release Study

The release of the factor was studied by keeping PHBV NPs loaded with TGF-β3 (ProSpec, Rehovot, Israel) at a concentration of 30 ng/mL in chondrogenic differentiation culture media (StemCell, Vancouver, BC, Canada) without the presence of cells for 28 days.

The process was based on sampling the supernatant every 24 h, except for the first 2 days, every 12 h. The supernatant was centrifuged at 1500× *g* for 5 min in a refrigerated microcentrifuge to remove any traces of NPs from the collected medium. The next step was to quantify the amount of TGF-β3 released into the medium by enzyme-linked immunoassay (ELISA) (Human TGF-β3 DuoSet ELISA; R&D Systems, Minneapolis, MN, USA) following the manufacturing instructions. Free TGF-β3 at a concentration of 30 ng/mL was used as a positive control, and the supernatant of the empty NPs was used as a negative control.

### 4.5. Biocompatibility of the Nanoparticles

The viability, proliferation, and chondrogenic differentiation of the primary cultures of the MSCs in the presence of NPs were performed to ensure the preclinical efficacy and safety of the new system. This capacity was evaluated in 2D (monolayer) and 3D (micromasses).

In the 2D experiment, approximately 30,000 primary MSCs were seeded per well in an eight-well chamber (SPL Life Sciences, Pocheon, South Korea). The cells were cultured with NPs loaded with TGF-β3 (TGF-β3 NPs) at a concentration of 30 ng/mL only at the beginning of the experiment in a chondrogenic differentiation medium (PromoCell, Heidelberg, Germany) with 1% P/S. The negative control was cells treated with empty NPs and cultured in a DMEM medium with 20% FBS and 1% P/S. The positive control was free TGF-β3 added twice a week to cells in a chondrogenic differentiation medium with 1% P/S to maintain the concentration at 30 ng/mL. These monolayer cultures were incubated for 21 days at 37 °C in a humid atmosphere of 5% CO_2_. Each process was performed in triplicate (n = 3).

After this, the wells were fixed with 4% paraformaldehyde, and immunofluorescence analysis was performed using anti-collagen type II ab34712 (Abcam, Cambridge, UK) and anti-collagen type I ab138492 (Abcam, Cambridge, UK) as the primary antibodies and rabbit anti-IgG (H + L) Alexa Fluor™ 594 cross-adsorbing antibody (Thermo Fisher, Waltham, MA, USA) as the secondary antibody. Counterstaining was performed with Bisbenzimide Hoechst 33342 (Merck, Darmstadt, Germany).

In the 3D experiment, micromasses formed with approximately 200,000 MSCs were used. The culture media used were the same as those in the 2D experiment. The conditions (TGF-β3 NPs, empty NPs, and free TGF-β3) were the same as in the 2D experiment. The 3D cultures were incubated for 21 days at 37 °C in a humid atmosphere with 5% CO_2_. In this case, the experiment with TGF-β3 NPs was conducted in triplicate (n = 3), and positive and negative controls were included as single-replicate reference conditions (n = 1).

After 21 days, the micromasses were fixed for 24 h in 4% paraformaldehyde, dehydrated with different volumes of ethanol, and embedded in paraffin to be cut on a Leica RM2255 microtome (Leica Biosystems, Nussloch, Germany). For the histological evaluation of the micromasses, the sections were deparaffinized in xylol and rehydrated in decreasing concentrations of ethanol. Histological analysis of the sections was performed with SO and MT stains to detect the presence of proteoglycans and to assess cell morphology and the presence of collagen, respectively.

All the samples were observed on an Olympus BX61 microscope (Olympus Corporation, Tokyo, Japan) coupled to an Olympus DP71 (Olympus Corporation, Tokyo, Japan) digital camera. The immunofluorescence was detected using the CoolLED pE-300 Series LED Illumination System (version 1.16, CoolLED, Andover, UK). Images were taken with CellSens Dimension software 4.1 (Olympus Corporation, Tokyo, Japan). ImageJ software (version 1.54p, National Institutes of Health, Bethesda, MD, USA) was used for image analysis to quantify the color intensity of each staining.

### 4.6. Generation of Chondrogenic Constructs

For the generation of chondrogenic constructs, 3D cultures on Col I scaffolds of 6 mm in diameter with approximately 250,000 MSCs were used. To study the chondrogenic effect of the NPs and their ability to generate chondrogenic constructs in vitro, TGF-β3 NPs were added only at the beginning of the experiment to the chondrogenic differentiation medium with 1% P/S at a concentration of 30 ng/mL. Cell-free scaffolds and scaffolds with MSCs and empty NPs cultured in the same medium were used as negative controls. As a positive control, a scaffold with MSCs cultured with free TGF-β3 was added twice a week to maintain the concentration in the chondrogenic differentiation medium with 1% P/S at the stipulated 30 ng/mL. In this case, the experiment with TGF-β3 NPs was conducted in quintuplicate (n = 5), and positive and negative controls were included as single-replicate reference conditions (n = 1).

After 30 days of culture, the constructs were fixed for 24 h in 4% paraformaldehyde, dehydrated with different volumes of ethanol, and embedded in paraffin to be cut in the microtome. Finally, histological analysis of these sections was carried out with SO and MT. The samples were observed under the Olympus microscope, and images were taken with CellSens Dimension software.

### 4.7. Ex Vivo Repair Model

An ex vivo repair model of focal human articular cartilage lesions was developed using samples from the A Coruña Tissue Bank. The articular cartilage samples were biopsied with a 6 mm punch, where a focal lesion of 3 mm in diameter was created. At the same time, chondrogenic constructs were generated in a 4 mm Col I sponge with approximately 250,000 MSCs. After at least one hour incubated at 37 °C in a humid atmosphere with 5% CO_2_, the constructs were placed in the cartilage lesions. Encapsulated TGF-β3 (TGF-β3 NPs) was added at the beginning of the experiment to the chondrogenic differentiation medium with 1% P/S at a concentration of 30 ng/mL. Negative controls were tested in empty lesions and lesions with constructs but treated with empty NPs cultured with a DMEM medium with 20% FBS and 1% P/S. The positive control was a construct treated with free TGF-β3 added to the chondrogenic differentiation medium with 1% P/S twice a week to obtain a constant concentration of 30 ng/mL. These models were kept in the oven at 37 °C in a humid atmosphere with 5% CO_2_ for one and a half months. The experiment with TGF-β3 NPs was conducted in triplicate (n = 3). Positive and negative controls were tested in duplicate (n = 2).

For the histochemical and immunohistochemical evaluation, the ex vivo repair model was fixed and embedded in paraffin. Only an ECM formed, and the adhesion areas of the construct in the cartilage lesion were evaluated, but not the cartilage itself. MT and SO stains were used for the histochemical evaluation. Immunohistochemical analyses were performed by incubating the deparaffinized sections with the following primary antibodies: anti-collagen type II ab34712 (Abcam, Cambridge, UK), anti-collagen type I ab138492 (Abcam, Cambridge, UK), aggrecan monoclonal antibody (BC-3) MA3-16888 (Thermo Fisher, Waltham, MA, USA), and anti- proliferating cell nuclear antigen (Ab-1) mouse mAb (PC10) (Merck, Darmstadt, Germany). To determine the antigen–antibody interaction, the EnVision Detection Systems Peroxidase/DAB, Rabbit/Mouse K5007 kit (Agilent, Santa Clara, CA, USA) was used.

The quality of the repair tissue in this ex vivo model was measured using the modified ICRSII semi-quantitative scale, considering only those parameters that can be observed in this type of model. That means that in this case, only six of the fourteen parameters initially described were assessed [45]. Tissue morphology and matrix staining were quantitatively analyzed based on two parameters: (1) the percentage of stained area relative to the total defect area, and (2) the normalized integrated density normalized to the negative control group treated with empty NPs. To combine both parameters into a unified score, a grading system from 0 to 4 was applied based on the percentage of stained area (0–24% = 1; 25–49% = 2; 50–74% = 3; 75–100% = 4). In parallel, normalized integrated density values were divided into tertiles and assigned a score from 1 to 3. The two scores were then multiplied to obtain a final combined score ranging from 1 to 12. This final score was used to estimate the degree of tissue repair according to the ICRS II criteria. Cell morphology was evaluated using the Trainable Weka Segmentation plugin in ImageJ, a machine-learning tool that identifies relevant and non-relevant regions based on user-defined training examples. The segmented regions were then used to quantify the area occupied by cells with a cartilage-like morphology. Chondrocyte clustering was not quantified due to the absence of visible clusters in the samples. The surface architecture and basal integration were assessed through blind evaluation by three independent experts in tissue engineering and cell therapy. The scores obtained from all these parameters were combined to generate a total score reflecting the overall quality of the lesion repair.

### 4.8. Statistical Analysis

All statistical analyses were performed using GraphPad Prism 8.0.2. Data are presented as mean ± standard error of the mean (SEM), except for the cytotoxicity assay, in which results are expressed as mean ± standard deviation (SD). Normality was assessed using the Shapiro–Wilk test. Significance levels were defined as follows: *** *p* < 0.01; ** *p* < 0.05; * *p* < 0.1.

In the 2D biocompatibility experiment, data passed the normality test; therefore, group comparisons were performed using one-way analysis of variance (ANOVA), followed by Tukey’s multiple comparisons test. In the ex vivo model, the TGF-β3 NP condition was tested in triplicate (n = 3), and the positive and negative controls (free TGF-β3 and empty NPs) were tested in duplicate (n = 2). Due to the limited sample size, a non-parametric approach was selected. Statistical analysis was performed using the Mann–Whitney U test, followed by Dunn’s multiple comparisons test. For analyses involving normalized integrated density values, statistical tests were performed using the raw (non-normalized) data to ensure the validity of the analysis. The normalized values were used exclusively for graphical representation and comparative visualization.

## 5. Conclusions

In this project, a study was carried out on the applicability of whey-derived PHBV NPs as a DDS for TGF-β3 for use as a possible biological therapy for OA. From the in vitro study of the cytotoxicity of the biopolymer, it was concluded that two of the studied materials, purified with chloroform and 86% ethanol, were optimal for use in biomedicine, as they did not reduce cell viability by more than 30%. From the release study, it can be concluded that the NPs were able to maintain a sustained release over time. Cell viability, proliferation, and differentiation studies showed that MSCs can grow and differentiate into chondrocytes in the presence of NPs and synthesize an ECM with proteoglycans and collagen. Finally, the ex vivo repair model showed that NPs with a single initial encapsulation of TGF-β3 achieved higher or comparable results to the positive control, which received two doses per week, demonstrating their efficiency as a DDS.

In summary, the main objective of evaluating the functionality of NPs produced from dairy by-products and developing a DDS capable of overcoming key limitations, such as the need for a single delivery method to mitigate the problems associated with multiple high doses of TGF-β3, was successfully achieved.

## Figures and Tables

**Table 1:** Free transforming growth factor β3 (TGF-β3) added twice a week, empty nanoparticles, and TGF-β3 nanoparticles added at the beginning of the experiment.

Parameters	Empty Nanoparticles	TGF-β3 Nanoparticles	Free TGF-β3
Tissue morphology	8%	38%	13%
Matrix staining	8%	45%	25%
Cell morphology	1%	14%	3%
Chondrocyte clustering	100%	100%	100%
Surface architecture	75%	95%	85%
Baseline integration	85%	93%	80%
Total score	46%	64%	51%

## Data Availability

Data is contained within the article and Supplementary Materials.

## Supplementary Materials

The following supporting information can be downloaded at: https://www.mdpi.com/article/10.3390/ijms26114997/s1.

## Abbreviations

MTT	3-(4,5-dimethylthiazol-2-yl)-2,5-diphenyltetrazolium bromide
ACAN	Aggrecan
BM	Bone marrow
CO_2_	Carbon dioxide
Col I	Collagen I (biomaterial)
COL I	Collagen I (protein)
COL II	Collagen II (protein)
CHUAC	Complejo Hospitalario Universitario de A Coruña
COX-2	Cyclooxygenase 2
DDS	Drug delivery system
DMEM	Dulbecco’s Modified Eagle’s Medium
ELISA	Enzyme-linked immunoassay
ECM	Extracellular matrix
FBS	Fetal bovine serum
FDA	Food and Drug Administration
HB	Hydroxybutyrate
HV	Hydroxyvalerate
PCNA	Proliferating cell nuclear antigen
IL-1	Interleukin 1
IL-1β	Interleukin 1-beta
IL-6	Interleukin 6
MT	Masson’s Trichrome
MMP	Matrix metalloproteinase
MSCs	Mesenchymal stem cells
NPs	Nanoparticles
NO	Nitric oxide
NF-κB	Nuclear factor-kappaB
iNOS	inducible nitric oxide synthase
OA	Osteoarthritis
P/S	Penicillin/Streptomycin
PHBV	Poly (hydroxybutyrate-co-hydroxyvalerate)
PHA	Polyhydroxyalkanoate
PHB	Polyhydroxybutyrate
PP	Polypropylene
PGE2	Prostaglandin E2
SO	Safranin O
SED	Sociedad Española del Dolor
SEMDOR	Sociedad Española Multidisciplinar de Dolor
3D	Three dimensions
TGF-β3	Transforming growth factor β3
TNF-α	Tumor necrosis factor
2D	Two dimensions
