# Supplementary material for: Encapsulation of Transforming Growth Factor-β3 in Poly(hydroxybutyrate-co-hydroxyvalerate) Nanoparticles for Enhanced Cartilage Tissue Engineering
Source: Int J Mol Sci. 2025 May 22;26(11):4997. doi: 10.3390/ijms26114997 (PMC12154055; doi:10.3390/ijms26114997)
Supplement: Supplementary file 1 [file ijms-26-04997-s001.zip › ijms-3604154-supplementary.pdf]

# SUPPLEMENTARY DATA

# Encapsulation of transforming growth factor-β3 in poly (hydroxybutyrate-co-hydroxyvalerate) nanoparticles for enhanced cartilage tissue engineering

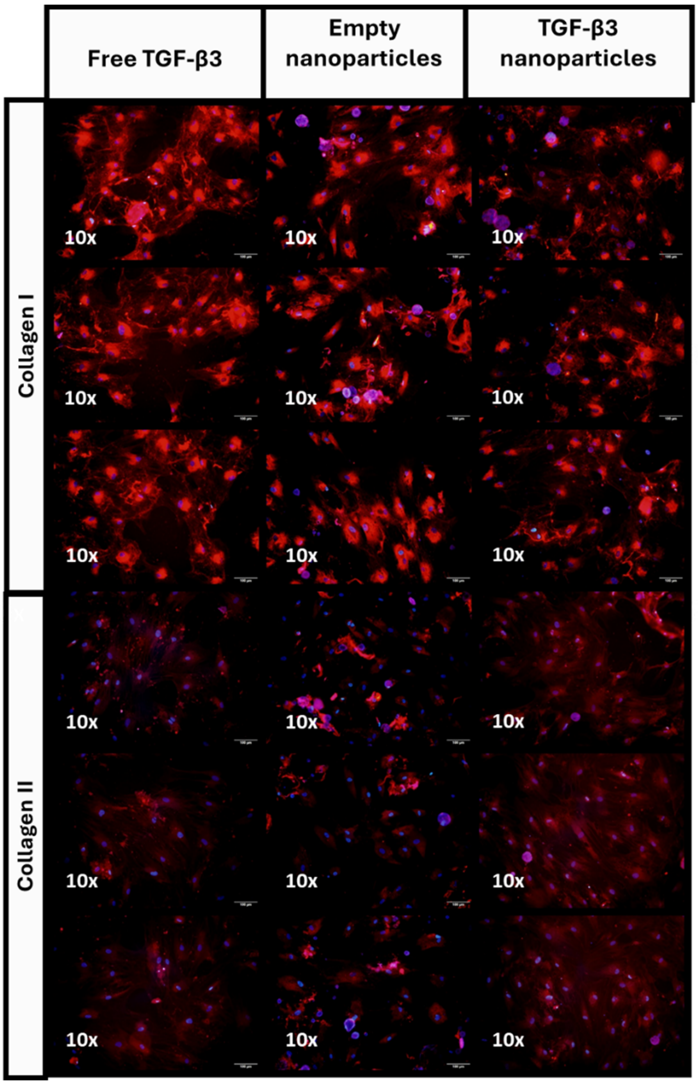


**Figure S1.** Immunofluorescence images of cells cultured for 21 days under different conditions: treatment with free TGF-β3 added twice a week, empty NPs, and TGF-β3 loaded NPs at the beginning of the experiment. Immunofluorescence analysis was performed using anti-collagen type II ab34712 (Abcam, UK) and anti-collagen type I ab138492 (Abcam, UK) as primary antibodies and rabbit anti-IgG (H+L) and Alexa Fluor™ 594 cross-adsorbing antibody (Thermo Fisher, USA) as secondary antibody. Scale bar represents 100 μm.

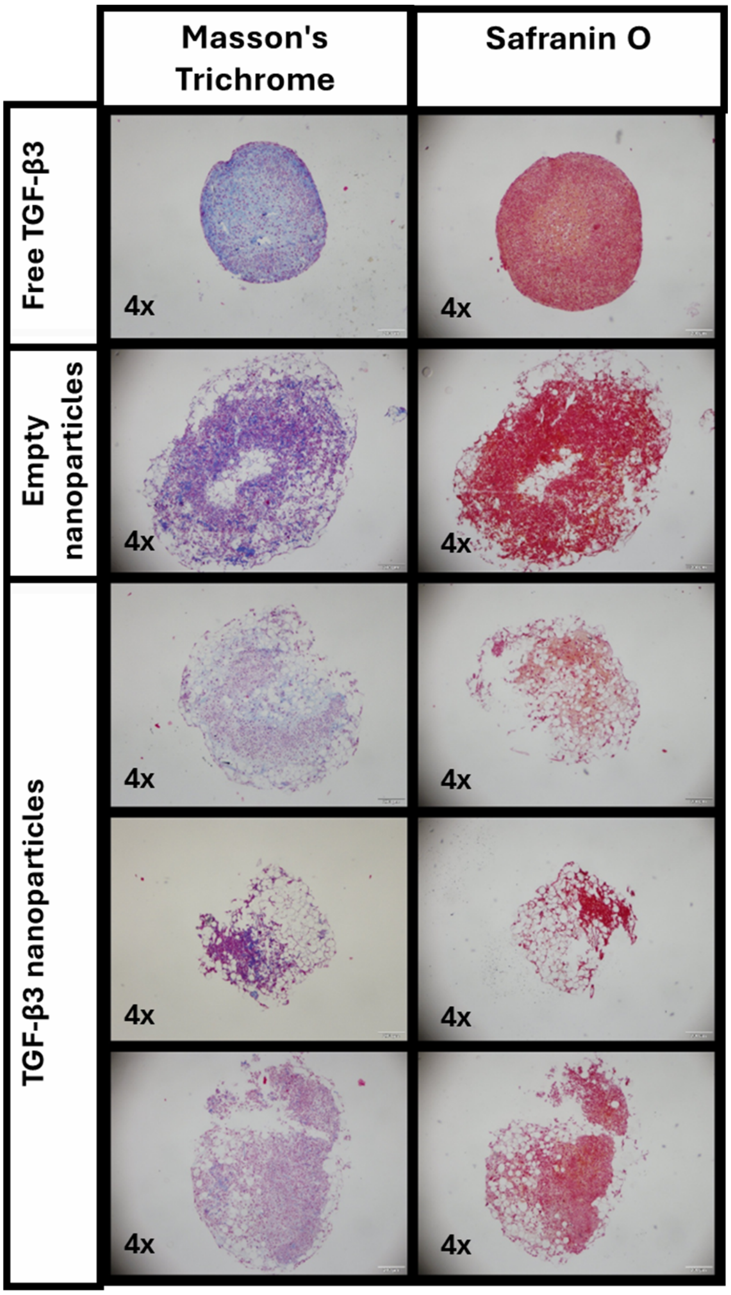


**Figure S2.** Histological staining of Masson´s Trichrome and Safranin O of micromasses cultured for 21 days under different conditions: treatment with free TGF-β3 added twice a week, TGF-β3 NPs at the beginning of the experiment, and empty NPs. Scale bars represent 200 μm.

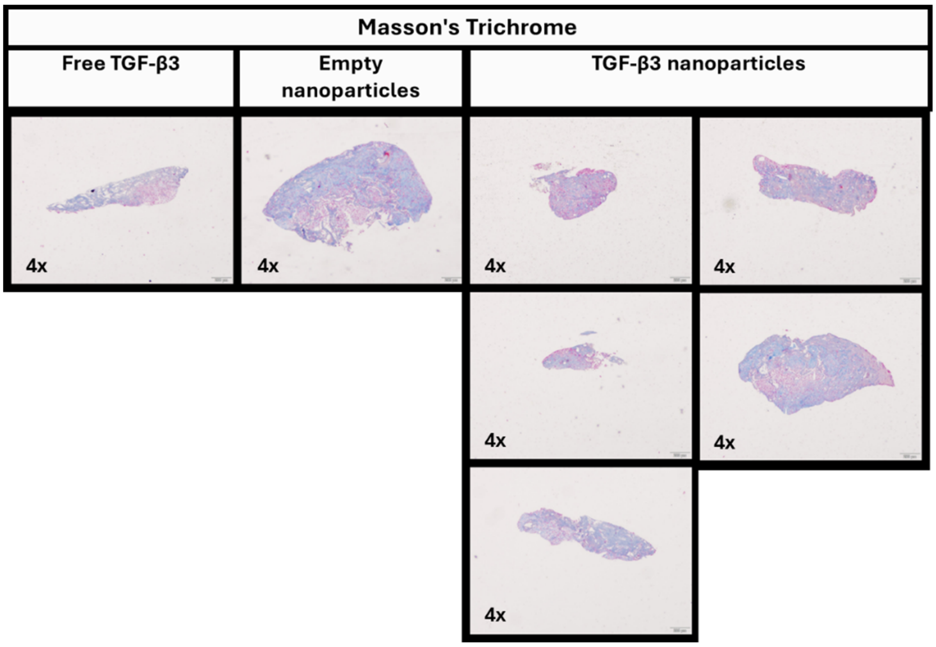


**Figure S3.** Histological Masson´s Trichrome staining of chondrogenic constructs cultured for 30 days under different conditions: treatment with free TGF-β3 added twice a week, TGF-β3 loaded NPs at the beginning of the experiment, and empty NPs. Scale bars represent 200 µm.

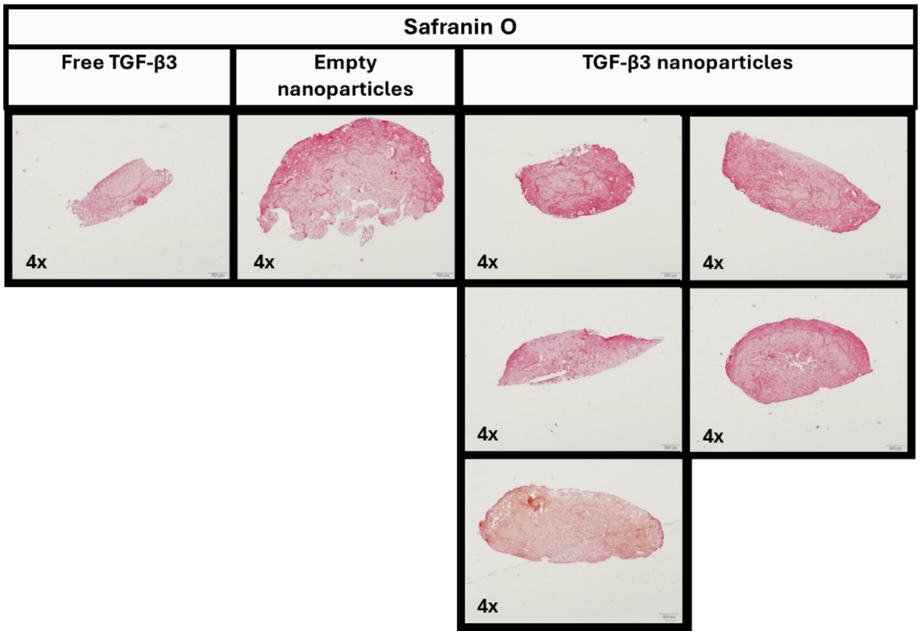


**Figure S4.** Histological Safranin O staining of chondrogenic constructs cultured for 30 days under different conditions: treatment with free TGF-β3 added twice a week, TGF-β3 loaded NPs at the beginning of the experiment, and empty NPs. Scale bars represent 200 µm.
